# Influence of Acute Exercise on DNA Repair and PARP Activity before and after Irradiation in Lymphocytes from Trained and Untrained Individuals

**DOI:** 10.3390/ijms20122999

**Published:** 2019-06-19

**Authors:** Maria Moreno-Villanueva, Andreas Kramer, Tabea Hammes, Maria Venegas-Carro, Patrick Thumm, Alexander Bürkle, Markus Gruber

**Affiliations:** 1 Molecular Toxicology Group, Department of Biology, Box 628, University of Konstanz, 78457 Konstanz, Germany; tabea.hammes@uni-konstanz.de (T.H.); alexander.buerkle@uni-konstanz.de (A.B.); 2 Human Performance Research Centre, Department of Sport Science, Box 30, University of Konstanz, 78457 Konstanz, Germany; andreas.kramer@uni-konstanz.de (A.K.); maria.venegas@uni-konstanz.de (M.V.-C.); p4trickthumm@gmail.com (P.T.); m.gruber@uni-konstanz.de (M.G.)

**Keywords:** aerobic fitness, endurance exercise, DNA strand breaks, radiation protection, PARP1 activity

## Abstract

Several studies indicate that acute exercise induces DNA damage, whereas regular exercise increases DNA repair kinetics. Although the molecular mechanisms are not completely understood, the induction of endogenous reactive oxygen species (ROS) during acute exhaustive exercise due to metabolic processes might be responsible for the observed DNA damage, while an adaptive increase in antioxidant capacity due to regular physical activity seems to play an important protective role. However, the protective effect of physical activity on exogenously induced DNA damage in human immune cells has been poorly investigated. We asked the question whether individuals with a high aerobic capacity would have an enhanced response to radiation-induced DNA damage. Immune cells are highly sensitive to radiation and exercise affects lymphocyte dynamics and immune function. Therefore, we measured endogenous and radiation-induced DNA strand breaks and poly (ADP-ribose) polymerase-1 (PARP1) activity in peripheral blood mononuclear cells (PBMCs) from endurance-trained (maximum rate of oxygen consumption measured during incremental exercise V’O_2max_ > 55 mL/min/kg) and untrained (V’O_2max_ < 45 mL/min/kg) young healthy male volunteers before and after exhaustive exercise. Our results indicate that: (i) acute exercise induces DNA strand breaks in lymphocytes only in untrained individuals, (ii) following acute exercise, trained individuals repaired radiation-induced DNA strand breaks faster than untrained individuals, and (iii) trained subjects retained a higher level of radiation-induced PARP1 activity after acute exercise. The results of the present study indicate that increased aerobic fitness can protect immune cells against radiation-induced DNA strand breaks.

## 1. Introduction

Regular physical exercise has many health benefits including greater longevity and a reduced risk of cardiovascular disease (CVD), and in particular, coronary heart disease (CHD), stroke, and cancer [1]. The V’O_2max_, also defined as maximal oxygen uptake, is the maximum amount of oxygen that the body is able to consume when exercising. Recent data suggest that both endurance training and high-intensity interval training (HIT) improve V’O_2max_ of healthy, young to middle-aged adults (for review see [2]). Endurance exercise, including high-intensity training, has considerable benefits in healthy young and elderly individuals; for example, it has been argued that it improves cardiorespiratory fitness, promotes longevity, slows down aging [3], attenuates age-related reduction in muscle strength [4], and improves visuospatial, verbal memory, and concentration performance in young healthy adults [5]. Furthermore, resistance exercise provides astronauts with an effective protection against decreases in body mass, muscle strength, bone mass, and aerobic capacity [6]. However, acute exercise is also known to induce oxidative stress, inflammation, and muscle fatigue [7]. At the molecular level, an acute bout of intense exercise induces transitory DNA damage, which is normally repaired within 24–72 h [8]. Several studies have specifically investigated the connection between exercise and DNA repair, suggesting an enhanced DNA repair through regular exercise [9] and a protective effect against radiation-induced DNA damage [10,11,12]. In the present study, we evaluated similar parameters in lymphocytes derived from trained versus untrained individuals.

### 1.1. Radioprotective Effect of Exercise

Recently, effects of physical activity on radiation-induced double-strand breaks have been investigated in a mouse model. Bone marrow cells isolated from acutely exercised, exercise-trained, and sedentary mice were irradiated *ex vivo* with 1 and 2 Gy. Exercise training significantly attenuated γH2AX foci formation in response to radiation [11]. Similar findings were reported in a human study. Blood samples from trained and untrained subjects were exposed *ex vivo* to X-irradiation before and after acute exercise and chromosomal damage was investigated. The basal level of chromosomal damage in lymphocytes remained the same in trained and untrained subjects while the X-ray-induced chromosomal damage was significantly increased in the untrained group, but not in the trained group [12]. In another more recent and extensive human study including about 100 participants, physical activity was found to be unrelated to DNA damage but was associated with increased repair at 60 min but not at 15 min after *ex vivo* irradiation [10]. These findings suggest that exercise training might induce an adaptive response to oxidative stress, protecting cells against radiation.

DNA single-strand breaks (SSBs) are by far the most frequent form of DNA damage, arising at a frequency of tens of thousands per cell per day [13]. Ionizing radiation induces DNA strand breaks that are rapidly recognized by PARP1 leading to its activation [14,15]. Activated PARP1 synthesizes poly (ADP-ribose) (PAR) using nicotinamide adenine dinucleotide (NAD) as a substrate [16,17]. After DNA damage, PARP1 is responsible for approximately 90% of the total cellular PAR formation and therefore the amount of PAR represents PARP1 activity [18]. Activation of PARP1 in response to DNA damage is one of the earliest cellular responses to genotoxic stress [19] triggering the recruitment of DNA repair factors [20,21]. Thus, PARP1 upregulation may lead to increased DNA repair of reactive oxygen species (ROS)-mediated DNA damage. Considering the crucial role of PARP1 in the repair of ROS-induced DNA damage, it is plausible to hypothesize that PARP1 may be a key component of the protective effects of exercise. Indeed, in a recent study, PARP1 has been shown to respond to exercise. PARP1 protein levels of the muscle *vastus lateralis* do not change in young trained but increase in young untrained individuals in response to an acute exercise bout [22].

### 1.2. ROS as Potential Mediator of Exercise-Induced DNA Damage

Regular exercise might enhance cellular protection against oxidative stress. Radiation induces DNA strand breaks mainly through ROS as a product of the radiolysis of water [23]. Thus, exercise training may counteract the effects of radiation by increasing the cellular antioxidant capacity. Furthermore, it is known that acute exercise can induce DNA damage. However, the underlying molecular mechanisms are not completely understood. Production of ROS, directly in the mitochondria or indirectly by cytokines or catecholamines during exercise, might play an important role in modulating the cellular acute and adaptive responses to exercise-induced DNA damage. Acute physical exercise induces formation of ROS, reactive nitrogen species (RNS), and the related oxidative damage [24]. Interestingly, moderate ROS formation during exercise can increase muscle force by modulating the contractile function of respiratory and limb skeletal muscle, while intense exercise can increase ROS production to a critical level, contributing to the development of acute muscle fatigue [25]. The extent to which reactive species are beneficial or detrimental depends on the exercise duration and intensity as well as the physical fitness of an individual [26]. Belviranlı and Gökbel reviewed the effect of physical exercise on oxidative status, concluding that acute exercise performed by untrained individuals increases oxidant levels and oxidative stress, but long-term exercise increases the activity of antioxidant enzymes, thus reducing oxidant production [27].

Apart from mitochondrial ROS, catecholamine and cytokines released during exercise could also contribute to ROS formation and in turn to DNA damage. During an acute bout of exercise, the human body reacts with the release of stress hormones [28]. For instance, after an exhaustive treadmill exercise test, the level of plasma catecholamines (epinephrine and norepinephrine) increases [29]. In turn, catecholamines can increase DNA damage; it has, for example, been shown that epinephrine induces DNA strand breaks in human leukocytes [30]. Exercise also initiates a cascade of inflammatory events. During and after acute exercise, cytokines released from muscle (myokines) mediate metabolic and inflammatory processes [31].

### 1.3. Effects of Exercise on DNA Repair

Inflammatory mediators (e.g., triggered by acute exercise) can directly affect the DNA repair process by either enhancing or repressing DNA repair. Several studies suggest that chronic inflammation may promote mutagenesis by inducing DNA damage and altering the expression of repair proteins [32]. In response to these detrimental acute effects of some bouts of exercise, cellular adaptations after long-term training interventions have been observed. For instance, adaptive response to regular exercise counteracts the age-associated increase in 8-OHdG levels, and increases the DNA repair [33]. Furthermore, exercise training induces an increased activity of mitochondrial 8-oxoguanine DNA glycosylase (OGG1), which is responsible for the excision of the mutagenic base byproduct 8-oxoguanine, in the nuclei of red fibers but a decreased activity in nuclei of white fibers [33]. Interestingly, the protective effects of exercise have been reported not only in skeletal muscle [34,35] but also in the liver [36,37], kidney [38], brain [39,40,41], and blood [42,43]. Regarding the protective potential of training on blood cells, DNA damage is lower in peripheral blood mononuclear cells of trained individuals compared to untrained individuals after exposure to oxidative stress [12].

The effects of acute exhaustive exercise seem to be detrimental, while the effects of long-term physical training might improve cellular defense mechanisms. Furthermore, the acute effects of exercise can be very different from the long-term effects. Although there are several studies reporting the effects of training on DNA damage and DNA repair, to our knowledge, there are no studies on the potential beneficial effect of endurance training versus acute exercise on radiation-induced PARP1 activity in human immune cells. Therefore, the aim of this study was to determine the overall effects of acute exhaustive exercise and physical fitness (aerobic capacity) on DNA damage, radiosensitivity, and radiation-induced PARP1 activity in immune cells isolated from healthy trained and untrained volunteers. We expected lower exercise-induced DNA strand breaks and higher radiation-induced PARP1 activity and enhanced DNA repair in immune cells from trained subjects when compared to untrained subjects.

## 2. Results

### 2.1. Aerobic Fitness Maintains Lower Endogenous PAR Levels and Protects Against Acute Exercise-Induced Endogenous DNA Strand Break Formation

**Figure 1:**
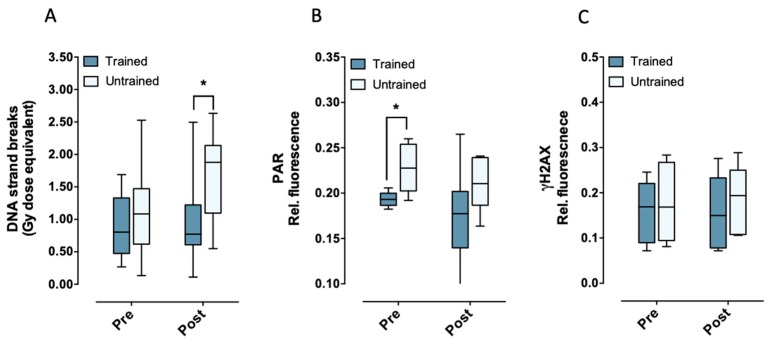
Endogenous DNA strand breaks (**A**), PAR (**B**), and γH2AX (**C**) measured in PBMCs from trained and untrained individuals before (pre) and after (post) exhaustive exercise. Statistical significance was calculated by students paired t-test (* *p* < 0.05). *n* = 15 trained and 15 untrained (**A**), 6 trained and 6 untrained (**B**,**C**) individuals. Rel. fluorescence signal of PAR and γH2AX represent the mean fluorescence normalized to the sum fluorescence intensity of the corresponding measured points.

We found no difference in endogenous DNA strand breaks between trained and untrained individuals before exercise (pre). However, there was a significantly (*p* = 0.0329) increased amount of endogenous DNA strand breaks after acute exercise (post) in untrained individuals (Figure 1A). Interestingly, following acute exercise, cells from subjects with high V’O_2max_ exhibited significantly lower endogenous DNA strand breaks than subjects with low V’O_2max_ (see below). Furthermore, basal PARP1 activity seemed to be higher in untrained subjects, however, it only reached statistical significance (*p* = 0.0293) before exercise (Figure 1B). In parallel, cells from subjects with high V’O_2max_ showed lower endogenous PARP1 activity, which was significant before exercise (see below). Taking together, these results suggest that aerobic fitness might be associated with lower endogenous PARP1 activity and might have a protective effect on exercise-induced DNA strand breaks. In order to investigate the formation of DNA double-strand breaks, the phosphorylation of the histone H2AX (γH2AX), a well-established biomarker for radiation-induced DNA double-strand breaks, was determined. The endogenous amount of γH2AX did not differ between trained and untrained, neither before nor after exhaustive exercise (Figure 1C), concluding that exercise or aerobic fitness did not induce endogenous γH2AX signaling.

### 2.2. Aerobic Fitness Enhances DNA Repair and Maintains PAR Levels after Radiation

**Figure 2:**
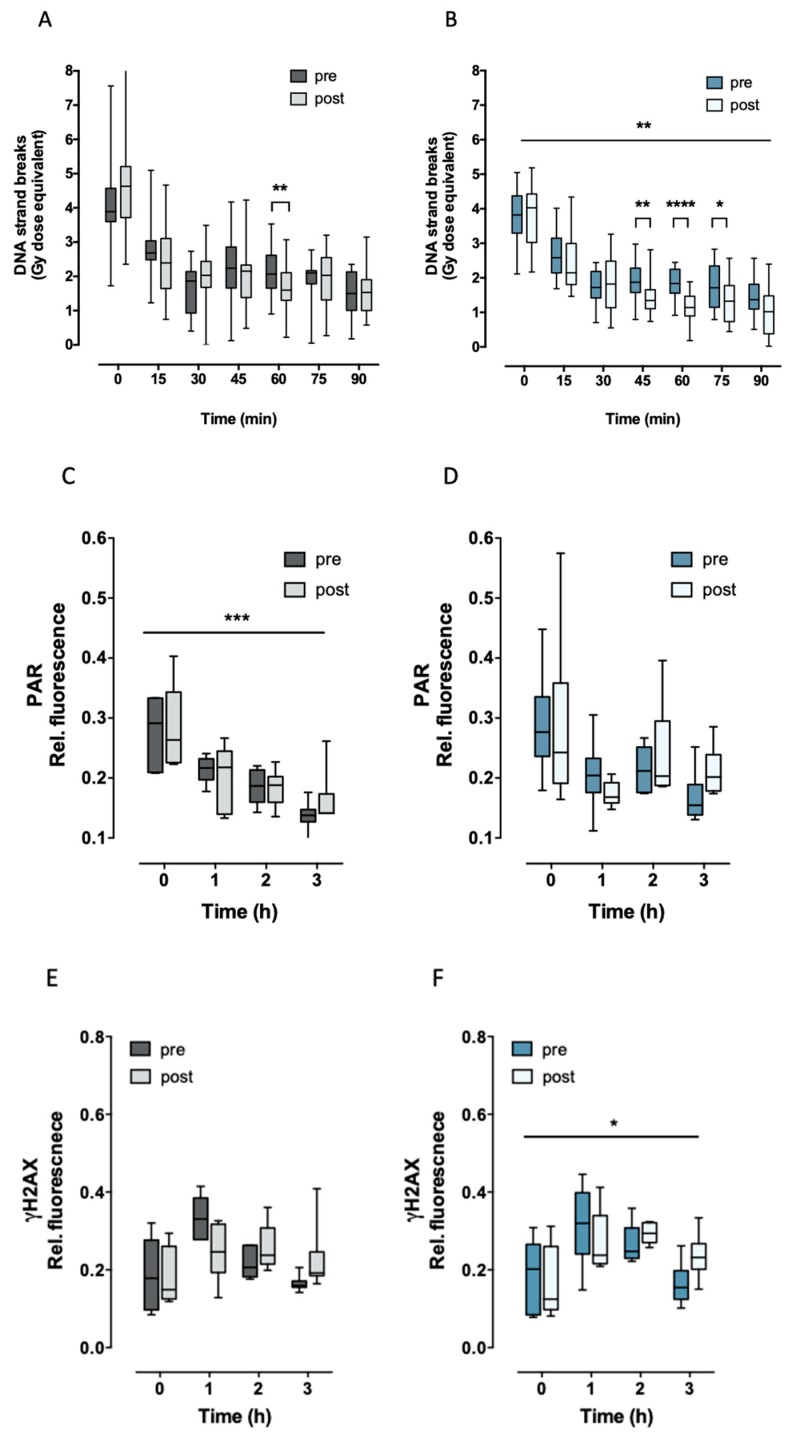
Radiation-induced DNA strand breaks (**A**,**B**), PARP1 activity (**C**,**D**), and γH2AX (**E**,**F**) were measured in PBMCs from trained (blue) and untrained (grey) individuals before (pre) and after (post) exhaustive exercise. PBMCs were irradiated on ice *ex vivo* with X-ray at a dose of 3.7 Gy (time point = 0 h) and thereafter incubated at 37 °C for the indicated time periods. Statistical significance was calculated by ANOVA samples matched by two factors (* *p* < 0.05). **A**,**B**: DNA repair kinetic was significantly different in trained individuals after workout (** *p* = 0.008). Sidak’s multiple comparisons test (post hoc test) showed significant differences between pre- and postworkout in untrained (repair time 60 min) and in trained individuals (repair times 45–75 min). **C**,**D**: PAR (pre and post) significantly decreased after radiation in untrained * *p* = 0.0004 but not in trained individuals). **E**,**F**: No significant difference was detected in γH2AX in untrained subjects when comparing pre- and postworkout, whereas trained individuals’ responses were significantly different after exhaustive exercise (training x workout interaction * *p* = 0.0269). Rel. fluorescence signal of PAR and γH2AX represent the mean fluorescence relative to fluorescence background. For γH2AX and PAR, 12 missing values in a total of 120 were replaced using the median of the corresponding values. Fifteen trained and 15 untrained subjects were analyzed for DNA strand breaks (DNA SB). Six trained and six untrained subjects were analyzed for PAR and six trained and six untrained subjects were analyzed for γH2AX. * *p* ≤ 0.05; ** *p* ≤ 0.01; *** *p* ≤ 0.001; **** *p* ≤ 0.0001.

**Figure 3:**
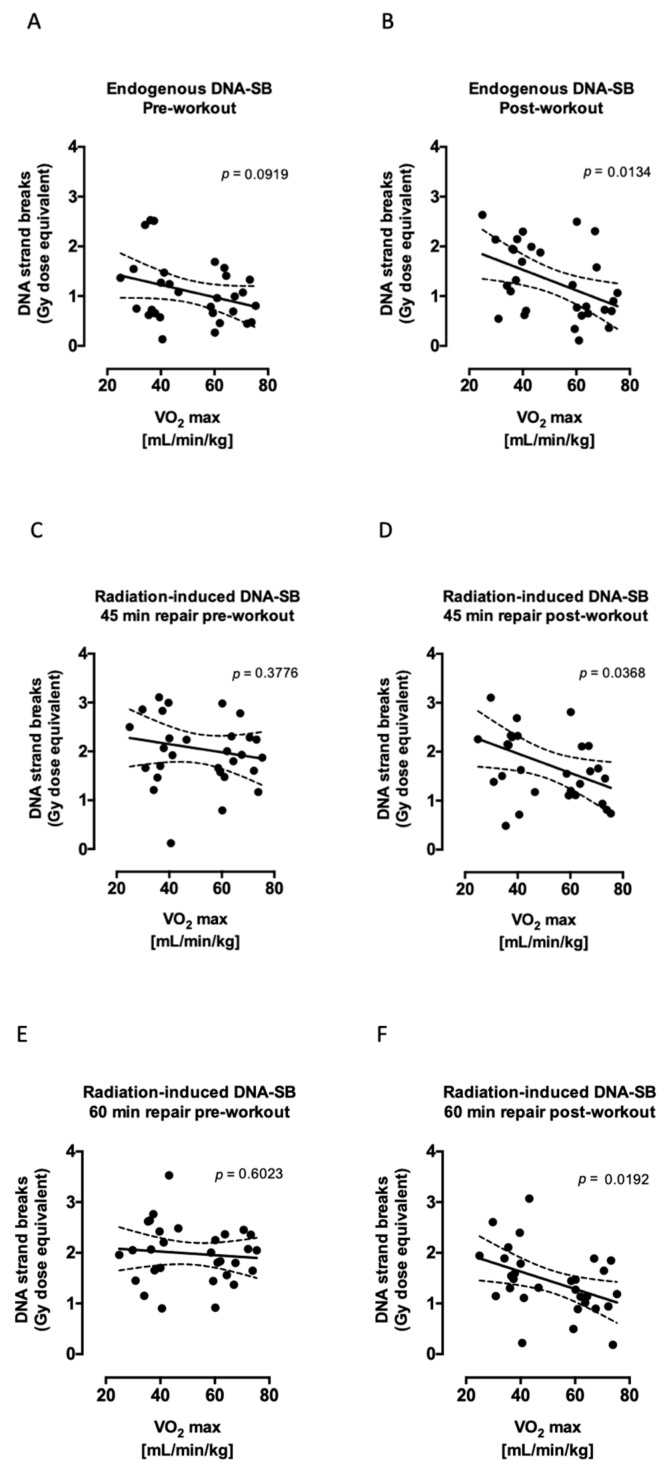

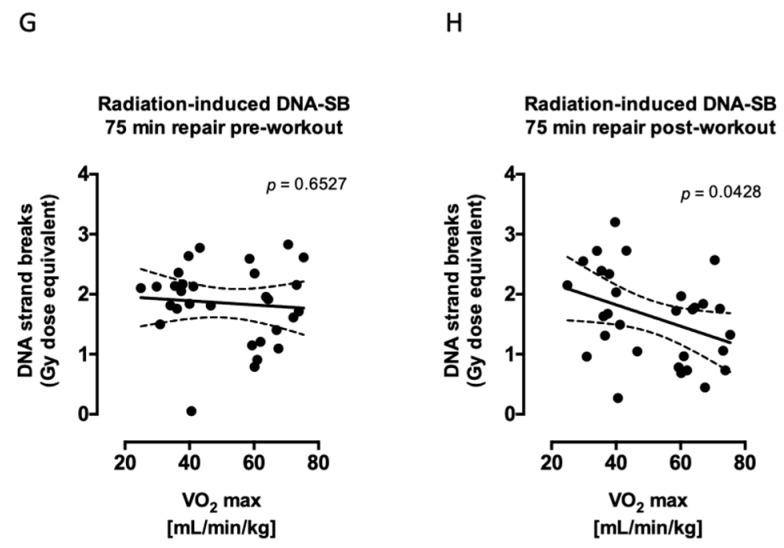
Correlations between endogenous (**A**,**B**) and residual (unrepaired) (**C**–**H**) DNA strand breaks with V’O_2max_. Cells were incubated for 45 (**C**,**D**), 60 (**E**,**F**), and 75 (**G**,**H**) min after irradiation. Linear regression shows a significant correlation between endogenous DNA strand breaks and V’O_2max_ as well as between DNA repair after exhaustive exercise (**B**,**D**,**F**,**H**) but not before (**A**,**C**,**E**,**G**). *n* = 30 subjects in each plot (15 trained and 15 untrained). Statistical significance means a significant linear relationship between x and y. The *p* values were calculated from an F test and are displayed in each figure.

Both groups, trained and untrained, showed a different DNA strand break repair kinetic before and after acute exercise (DNA repair x acute exercise interaction *p* < 0.0002). However, this difference was more pronounced in trained individuals (over all two-way ANOVA *p* < 0.005). The immediate (5–30 min) response to radiation did not show any significant difference between the groups, while the amount of DNA strand breaks repaired between 45–90 min of time was significantly higher in trained subjects (Figure 2A,B). This effect was also reflected in the correlation found between the residual (unrepaired) DNA strand breaks with maximal oxygen uptake capacity (V’O_2max_) after but not before exhaustive exercise. Cells from subjects with high V’O_2max_ exhibited significantly lower residual (at 45, 60, and 75 min after irradiation) DNA strand breaks than subjects with low V’O_2max_ (Figure 3). In parallel, 2–3 h after exercise, PARP1 activity was maintained slightly but significantly higher (*p* = 0.0455) in trained compared to untrained subjects (Figure 2C,D).

Previous studies report a maximal H2AX phosphorylation between 0.5 and 2 h after radiation exposure [44,45,46,47]. Similarly, in our experiments, the maximal H2AX phosphorylation was reached after 1–2 h postirradiation when compared to nonirradiated cells. The radiation-induced γH2AX signal increased in both trained and untrained subjects 1 h after irradiation. However, the overall formation and removal of γH2AX signal was significantly different after performing exhaustive exercise only in trained individuals (aerobic status x acute exercise interaction *p* = 0.0269). Furthermore, the levels of γH2AX 2–3 h postirradiation were maintained higher after intensive exercise by both trained and untrained individuals, indicating a higher radiation-induced γH2AX response due to one bout of intensive exercise (Figure 2E,F).

**Figure 4:**
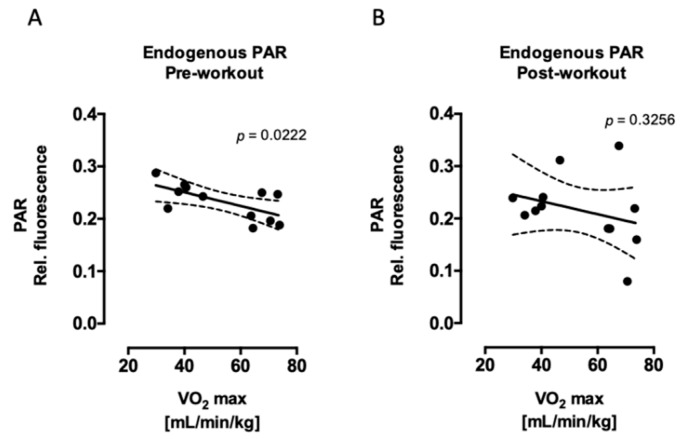

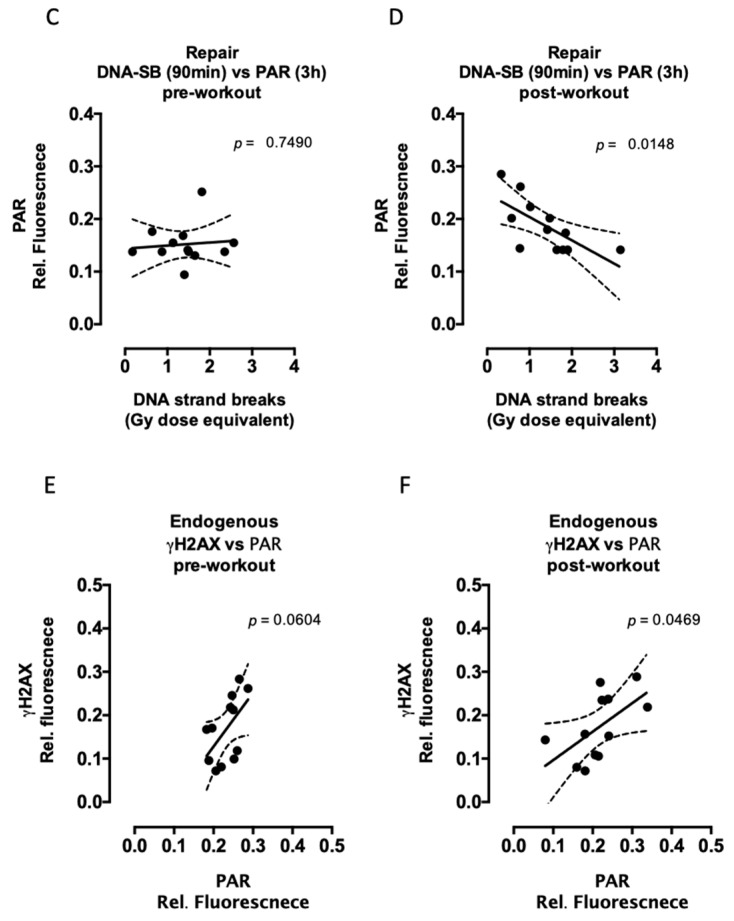
Correlations between endogenous PARP1 activity and V’O_2max_ (**A**,**B**), DNA strand breaks 90 min after radiation and PARP1 activity 3 h after radiation (**C**,**D**), and endogenous PARP1 activity and γH2AX (E,F). Linear regression shows significant negative correlation between PARP1 activity and V’O_2max_ before exercise (**A**) as well as between DNA repair and PARP1 activity after acute exercise (**D**). A positive significant correlation was found between PARP1 activity and γH2AX (**F**) after exercise but not before (**E**). *n* = 12 subjects in each plot (six trained and six untrained). Statistical significance means a significant linear relationship between x and y. The *p* values were calculated from an F test and are displayed in each figure.

Similar to DNA single-strand breaks, endogenous (without radiation) PARP1 activity was associated with aerobic training (Figure 4A,B), although this association was significant only before performing exhaustive exercise. This means that individuals with a higher basal PARP1 activity have a lower aerobic capacity. On the other hand, maintenance of PARP1 activity at 3 h after radiation was significantly negatively correlated with residual (90 min after radiation) DNA strand breaks after but not before performing exhaustive exercise (Figure 4C,D). This indicates that aerobic fitness is associated with PARP1 activity when individuals performed exhaustive exercise and, additionally, cells were challenged with radiation. Furthermore, endogenous PARP1 activity was associated with endogenous γH2AX signaling, this association was significant after acute exercise (Figure 4E,F).

**Figure 5:**
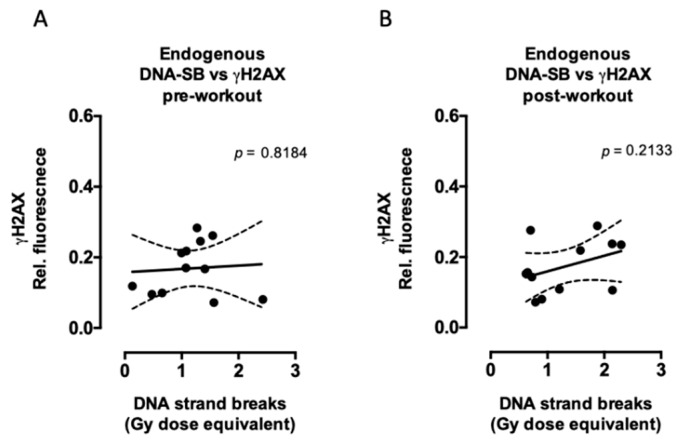

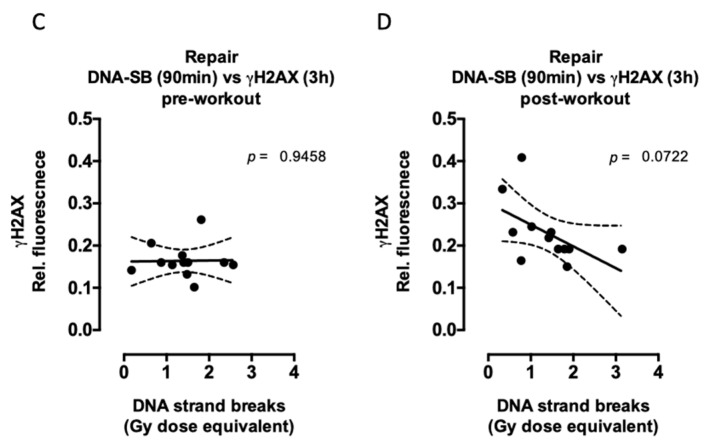
Correlations between endogenous (**A**,**B**) and radiation-induced (**C**,**D**) γH2AX and DNA strand breaks. No statistical significance was detected between γH2AX and DNA strand breaks either pre or post performing exhaustive exercise. *n* = 12 subjects in each plot (six trained and six untrained). Statistical significance means a significant linear relationship between x and y. The *p* values were calculated from an F test and are displayed in each figure.

The relationship between DNA strand breaks and γH2AX signaling was also evaluated (Figure 5). Although the correlations between the amount of DNA strand breaks and γH2AX signaling after exercise did not reach statistical significance, there was a tendency for a positive correlation between endogenous γH2AX and endogenous DNA strand breaks (Figure 5B), while after radiation, the residual γH2AX was negatively correlated with the residual DNA strand breaks (Figure 5D).

## 3. Discussion

In the present study, we showed that immune cells from endurance-trained individuals with a higher aerobic capacity (V’O_2max_ >55 mL/min/kg) repaired the *ex vivo* radiation-induced DNA strand breaks “faster” than untrained individuals with low aerobic capacity (V’O_2max_ <45 mL/min/kg) after the subjects performed exhaustive exercise. The mechanisms behind the protective effect of long-term exercise are not well understood. However, production of ROS generated during exercise could be considered a key player in the modulation of DNA damage response. ROS can interact directly with specific DNA promoters or activate transcription factors [48] such as nuclear factor (erythroid-derived 2)-like 2 (Nrf2), which regulates the expression of several antioxidant and detoxifying genes [49]. Additionally, ROS interact with ATM-regulated DNA damage response [50]. Moderate to high-intensity exercise can stimulate the adaptive responses by triggering endogenous antioxidant defense mechanisms against excessive ROS, thereby maintaining muscle redox balance [51] and enabling cells to cope with exposure to high levels of oxidants that would normally be toxic. Furthermore, emerging data identify regular physical exercise as one potential strategy for improving DNA repair capacity [9], thus enhancing the body’s capability to prevent and deal with diseases such as cancer. In order to enhance our understanding of the role of exercise in repairing DNA damage, we investigated the effect of acute exercise as well as aerobic fitness on the capacity of immune cells to repair radiation-induced DNA strand breaks and the role of PARP1 activity as a main player in initiating DNA repair.

We found a higher endogenous level of DNA strand breaks in untrained individuals after exhaustive exercise. This finding is in accordance with previous studies indicating the detrimental effect of an intensive workout on DNA, probably through the generation of oxidative stress [52,53]. Furthermore, trained individuals were protected against workout-induced DNA strand breaks. These results also support published data [54].

While endogenous DNA damage has been widely investigated, the effect of aerobic fitness on radiation-induced DNA damage has gained less attention. To our knowledge, only a few studies investigated the effect of training on radiation-induced DNA damage. Similar to our study, radiation-induced chromosomal damage was evaluated in trained and untrained subjects after acute exercise; the X-ray-induced chromosomal damage was significantly enhanced after treadmill running (85% of maximal oxygen uptake for 30 min) in the untrained group, but not in the trained group [12]. Therefore, training might also protect cells against environmentally induced DNA damage.

Interestingly, we did not detect differences in the first 30 min of repair, which is in accordance with observations by Cash and collaborators [10]. Different methods used for detection of DNA strand breaks showed an initial fast repair component of 5–6 min, followed by a component between 10 and 30 min after irradiation [55], and recently, a biochemical kinetic model showed fast and slow double-strand breaks repair components [56]. Likely, the first 30 min of DNA repair correspond to the repair of DNA single-strand breaks and oxidized DNA bases while double-strand breaks take 0.5–12 h to be repaired [57,58,59]. In accordance with previous studies, our results showed an increase in the phosphorylation of H2AX histone 1 h after radiation. Training did not have a significant effect on the amount of γH2AX. However, before acute exercise, the amount of radiation-induced γH2AX was reduced to almost the basal levels while after acute exercise, the level of γH2AX remained high, suggesting an exercise-associated slower DNA double-strand break repair independent of the subjects’ aerobic fitness. These data are not in accordance with published data obtained from mouse experiments where exercise training significantly attenuated γH2AX foci formation in response to 1 Gy radiation challenge [11]. The observed discrepancy could be explained by the radiation dose applied, which was much lower in the mouse study, or by the differences in the DNA repair kinetics between organisms. For instance, DNA repair is upregulated in long-lived humans compared with short-lived mice [60]. Although the γH2AX signal remaining high may suggest a higher number of DNA double-strand breaks, it can also be associated with a higher activity of the DNA damage sensor proteins ataxia-telangiectasia-mutated (ATM) or DNA-PK kinase activity. Both ATM and DNA-PK can phosphorylate the histone H2AX rapidly after irradiation [61]. Therefore, a higher γH2AX signal could be associated with a higher ATM and/or DNA-PK activity resulting in a “faster” DNA double-strand break detection. However, further experiments looking at longer recovery time periods and protein activity after radiation are necessary in order to address this question.

We also assessed PARP1 activity by measuring the amount of PAR formed after exercise and/or radiation. We found a higher endogenous level of PAR in untrained individuals. This difference could be explained by a training-associated oxidative protection leading to a lower amount of endogenously induced DNA strand breaks in trained subjects and therefore low maintained PARP1 activity. Our findings are also consistent with existing data on PARP1 protein levels that did not change in young trained individuals whilst there was a PARP1 increase in untrained individuals following exercise [22]. Contrary to the endogenous PAR level, we found that the level of radiation-induced PAR is higher in trained subjects. This higher PARP1 activity seems to be accompanied by a higher DNA repair capacity. Indeed, our data indicate that a decreased amount of DNA strand breaks 90 min after radiation is associated with maintained high PARP1 activity 3 h after radiation (Figure 4). On the one hand, PARP1 activity is crucial for DNA repair, but on the other hand, sustained PARP1 activity has a tremendous effect on the homeostasis of NAD^+^ and NADH metabolism, which is crucial for maintaining the antioxidant capacity of cells [62]. Furthermore, PARP1 is needed in the response of ATM to gamma irradiation [63] and may act as a nick sensor and recruit ATM to the damaged site [64], allowing H2AX phosphorylation and DNA double-strand breaks to be repaired. In fact, our data showed a significant positive correlation (*p* = 0.0469) between endogenous PARP1 activity and γH2AX after acute exercise (Figure 4F). However, PARP1 is not only a crucial element of DNA repair but also regulates cell survival, cell-death pathways, and other cellular functions including transcriptional regulation, telomere cohesion and mitotic spindle formation, intracellular trafficking, and energy metabolism [65].

Ionizing radiation induces both single- and double-strand breaks simultaneously, whereupon the amount of DNA single-strand breaks is substantially higher than the amount of double-strand breaks. Thus, we also questioned the relationship between DNA single-strand breaks repair and double-strand breaks repair after irradiation. Although our data did not reach significance, after acute exercise, there was a clear tendency for a negative association between DNA strand breaks and γH2AX after radiation. This means that individuals with high residual DNA strand breaks showed a lower γH2AX signal and *vice versa*. This could be interpreted as a preference of first repairing DNA single-strand breaks and thereafter the repair of double-strand breaks can take place. A recently published numerical model exploring DNA repair when DNA single-stranded breaks and DNA double-stranded breaks exist simultaneously concluded that DNA single-strand breaks are repaired preferentially, and single- and double-strand breaks can be repaired simultaneously after most of the single-strand breaks have been repaired [66]. However, further extensive research will be necessary in order to answer this question.

**Figure 6:**
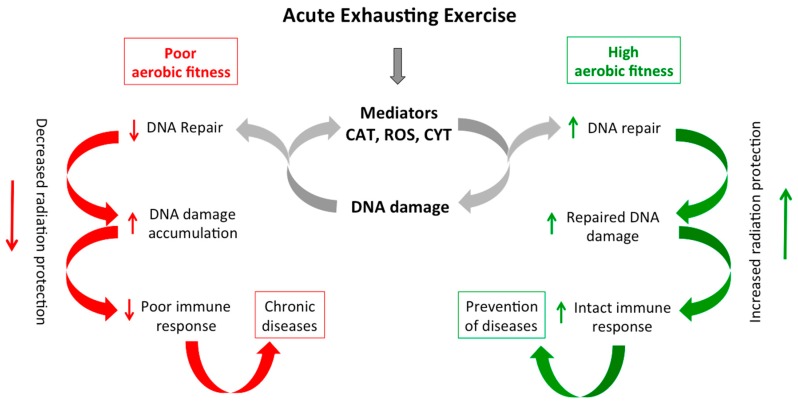
Cellular consequences of acute exhausting exercise in immune cells. One bout of acute exhaustive exercise induces DNA damage through mediators such as catecholamines (CAT), reactive oxygen species (ROS), and/or cytokines (CYT). Immune cells from individuals with poor aerobic fitness have a limited DNA repair capacity leading to accumulation of DNA damage and consequently a poor immune response (red arrows), while immune cells from individuals with a high aerobic fitness have an enhanced DNA repair capacity protecting immune system and therefore preventing the onset of diseases (green arrows).

Our results indicate a higher amount of rejoined DNA single-strand breaks after radiation in trained subjects and this is accompanied by higher PARP1 activity while higher H2AX phosphorylation was observed after acute exercise. The repair of radiation-induced DNA damage is essential to maintain genomic integrity. “Unrepaired” or “wrong-repaired” DNA single-strand breaks can affect cellular replication and transcription leading to genomic instability [67]. Furthermore, accumulation of unrepaired DNA single-strand breaks is associated with heart failure [68], genetic diseases [13], and cellular senescence [69], leading to serious biological consequences. Understanding the effect of aerobic fitness on radiation-induced DNA damage response could offer a new radioprotective strategy for decreasing the potentially harmful consequences of radiation exposure (Figure 6).

## 4. Materials and Methods

### 4.1. Experimental Design

Participants were recruited in accordance with the ethics committee of the University of Konstanz (approval #25/2018, 4 September 2018). Inclusion criteria: aerobic capacity (V’O_2max_) above 55 mL/min/kg or below 45 mL/min/kg (evaluated during an exhaustive ramp test on a cycle ergometer), male, age 20–36 years. Exclusion criteria: known heart conditions that preclude exhaustive exercise, previous positive testing for Hepatitis B, Hepatitis C, or human immunodeficiency virus (HIV), actuated cancer treatment or long-term treatment with glucocorticoids. Trained individuals (V’O_2max_ above 55 mL/min/kg) consisted of endurance-trained athletes, mostly racing cyclists, triathletes, and intermediate- to long-distance runners practicing an endurance sport, normally at a competitive level. Untrained individuals (V’O_2max_ below 45 mL/min/kg) lived a sedentary lifestyle without any regular exercise for the last three years. The exhaustive exercise consisted of a warm-up, three series of maximal vertical jumps, taps, and hops, and a ramp test until volitional exhaustion on a cycle ergometer. During the ramp test, the resistance of the cycle ergometer was gradually increased, requiring increasing power output by the participant. Directly before and 30 min after the exhaustive exercise session, about 60 mL of blood was drawn and peripheral blood mononuclear cells (PBMCs) were isolated as indicated in the section below. In order to stimulate DNA repair, PBMCs were irradiated with 3.7 Gy of X-ray *ex vivo*. The radiation-induced DNA strand breaks and PARP1 activity were measured immediately after radiation. To allow the DNA to repair, cells were incubated at 37 °C for several periods of time between 15 and 180 min.

### 4.2. Blood Draw

Venous blood was obtained from participants using monovettes containing a 3.2% citrate solution (S-Monovette^®^ 10 mL 9NC, Sarstedt) as anticoagulant. Peripheral blood mononuclear cells (PBMCs) were isolated from whole blood by Biocoll^®^ (Biochrome AG, Berlin, Germany) density gradient centrifugation following the manufacturer’s instructions. Cell concentration and viability were determined by using a CASY^®^ Counter.

### 4.3. DNA Single-Strand Breaks Detection

Several volumes of 100 µL (4 × 10^6^/mL) cell suspension (suspension buffer: 0.25 M meso-inositol; 10 mM sodium phosphate, pH 7.4; 1 mM magnesium chloride) were irradiated on ice using a biological X-ray Irradiator X-RAD 225 iX (Precision X-Ray, Inc, North Branford, CT, USA). The radiation time was 380 s at a dose rate of 0.59 Gy/min (70 kV, 30 mA, 70 cm distance, 1.25 mm Al filter) resulting in a total dose of 3.73 Gy. In order to allow DNA strand breaks repair cells were incubated at 37 °C for the indicated periods of time. DNA strand breaks were detected by the automated FADU assay [70,71], which has been successfully applied in numerous *in vivo* and in vitro studies [72,73,74,75,76,77,78,79,80,81,82,83,84,85]. This assay is based on alkaline DNA unwinding in a cell lysate under controlled conditions (time, pH, and temperature) staring at DNA strand breaks. SybrGreen^®^ (MoBiTec, Göttingen, Niedersachsen, Germany) was used as the marker for double-stranded DNA. The fluorescence signal was expressed as a measure of radiation dose (Gy-equivalent) using a published mathematical transformation [86].

### 4.4. PAR and γH2AX Detection

The detection of double-strand breaks and PARP1 activity was performed simultaneously using γH2AX and PAR-specific fluorescence-labeled antibodies. The fluorescence signal was detected by flow cytometry. DNA double-strand breaks trigger the phosphorylation of histone H2AX at serine 139. Therefore the level of phosphorylated histone H2AX (γH2AX) increases with increasing DNA DSB [87,88]. Under genotoxic stress poly (ADP-ribose) (PAR) is formed by PARP1. Accumulation of PAR was measured by flow-cytometry as established before [89]. Cells were resuspended in 400 µL of ice-cold FACS buffer (1× PBS, 0.5% FCS, 2 mM NaN_3_) and fixed by adding 400 µL of ice-cold (−20 °C) 100% ethanol. After 1 h at 4 °C, 900 µL of cold FACS buffer were added to the cell suspension and incubated at 4 °C for another 15 min. Cells were centrifuged (800× *g*, 5 min, at 4 °C) and the pellet was resuspended in cold FACS buffer and transferred to a 96-well plate (200 µL/well). The 96-well plate was centrifuged (750× *g,* 5 min, at 4 °C), supernatant was discarded, and the pellet was resuspended in 100 µL of FACS buffer containing the primary antibodies against γH2AX (Rabbit, Cell Signaling Technology, Germany; 1:400) and PAR (1:300; 1.18 mg/mL stock; 10H hybridoma cells from M. Miwa and T. Sugimura, Tokyo, Japan) and incubated at 4 °C overnight. Thereafter, fixed cells were centrifuged (750× *g,* 5 min, at 4 °C) and washed with 200 µL of FACS buffer. After centrifugation (750× *g*, 5 min, at 4 °C), cells were resuspended in 100 µL of FACS buffer containing both fluorophore-labeled secondary antibodies: Alexa Fluor^®^ 488 (1:1000; Cell Signaling Technology, Frankfurt am Main, Hessen, Germany) and Alexa Fluor^®^ 647 (1:1000; Cell Signaling Technology, Germany). After 45 min at 37 °C, the samples were centrifuged (750× *g*, 5 min at 4 °C) and washed twice with 200 µL of FACS buffer (750× *g*, 5 min at 4 °C). Cell pellet was resuspended in 200 µL of FACS buffer and measured in a flow cytometer (BD FACSVerseTM Flow Cytometer, Becton, Dickinson and Company, Franklin Lakes, NJ, USA). The samples were measured in a 96-well plate with the flow rate set to high, using the auto sampler of the flow cytometer. Samples were measured in triplicates. Data analyses were performed by FlowJo software (FlowJo, version 10.4.1, LLC, Ashland, OR, USA).

### 4.5. Exhaustive Bout of Exercise, Assessment of V’O_2max_

The main part of the bout of exhaustive exercise was the cycle ergometer test described below, which was also used to assess the participants’ V’O_2max_. Prior to the ramp cycle test, the participants’ height and weight were measured, and their body fat percentage was estimated using a seven-point skinfold caliper assessment (Harpenden Skinfold Caliper, Baty International, Burgess Hill, South East England, United Kingdom). After these anthropometric measurements, three exercises that require high power had to be completed: three maximal vertical jumps with the aim to jump as high as possible, two series of 10 repetitive hops each, and two tapping tests that required the participants to “sprint” on the spot with maximal effort for five seconds each. Each of these exercises was conducted with maximal effort and enough rest in between to fully recover in between trials.

Thereafter, we measured the maximal oxygen uptake (V’O_2max_; for an overview of V’O_2max_ testing, see e.g., [90]). Participants performed a ramp test on a cycle ergometer (Ergoselect 200, Ergoline, Bitz, Germany). After an initial 30 s of seated rest and a 3 min warm-up phase (40–80 W, depending on the ramp slope), subjects were instructed to start and maintain a constant cadence between 80 and 90 rpm while the load was continuously increased. To keep the test duration between 8 and 12 min, the ramp slope was set to 20 W/min, 25 W/min, 25 W/min, 30 W/min, or 35 W/min, based on body weight and training status. The load was increased and strong verbal encouragement was given until volitional exhaustion. Breath-by-breath oxygen uptake and carbon dioxide emission was monitored using the Ergostik system (Geratherm Respiratory GmbH, Bad Kissingen, Germany). The spirometry data was filtered by taking a moving average over 30 s. VO_2max_ was determined as the highest value obtained after filtering the data. We ensured that participants were indeed exhausted by monitoring heart rate and respiratory exchange ratio. All participants reached their maximal heart rate or a respiratory exchange ratio of >1.10 at the end of the ramp test.

## 5. Conclusions

In the present study, we revealed new insights into the effect of physical exercise on DNA damage and repair. Our results suggest that aerobic fitness can provide white blood cells (PBMCs) with more effective defense mechanisms against radiation. Besides the proven effectiveness of exercise to increase cardiovascular and musculoskeletal health, it might be effective in protecting humans from the detrimental effects of radiation-induced damage. This could have applications in the fields of radiation therapy and occupational radiation exposure. For instance, implementation of adequate exercise could protect pilots, astronauts, or nuclear plant employees that have been accidentally exposed to radioactive materials.
